# Data for the analysis of willingness to pay for Italian beaches

**DOI:** 10.1016/j.dib.2019.103815

**Published:** 2019-03-07

**Authors:** Ilaria Rodella, Fabio Madau, Massimiliano Mazzanti, Corinne Corbau, Donatella Carboni, Kizzi Utizi, Umberto Simeoni

**Affiliations:** a Department of Engineering, Ferrara University, Via Saragat 1, 44122 Ferrara, Italy; b Department of Science for Nature and Environmental Resources, Sassari University, Via Enrico de Nicola 1, 07100 Sassari, Italy; c Department of Economics and Management, Ferrara University, Via Voltapaletto 11, 44121 Ferrara, Italy; d Department of Physics and Earth Sciences, Ferrara University, Via Saragat 1, 44122 Ferrara, Italy; e Department of Human and Social Sciences, Sassari University, Via Roma 151, 07100 Sassari, Italy

## Abstract

The data presented herein relates to the article entitled “Willingness to pay for management and preservation of natural, semi-urban and urban beaches in Italy” [1].

Data of several Italian beaches are collected considering shape, anthropogenic characteristics, use, activity and urbanization levels. Descriptive statistics of beach characteristics and beach users are presented, on the basis of about 5000 interviews.

**Table:** Specifications table

Subject area	*Earth Science - Economics*
More specific subject area	*Coastal management*
Type of data	*Table and graph*
How data was acquired	*Survey*
Data format	*Analyzed*
Experimental factors	*The data were analyzed by various beach features and demographic strata (residency, age, sex, education, income).*
Experimental features	*The relationship between beach features, demographic characteristics and the Willingness to Pay (WTP) of beach-goers were determined.*
Data source location	*Data are available for forty-one localities of eleven regions in Italy* (see also Supplementary material 2 and interactive kmz map): 1 *Lido di Venezia, Rosolina Mare (Veneto region)* 2 *Lido di Nazioni, Lido di Pomposa, Lido degli Scacchi, Porto Garibaldi (Emilia-Romagna region)* 3 *Porto Recanati, Civitanova Marche, Porto Sant'Elpidio, San Benedetto Del Tronto (Marche region)* 4 *Manfredonia, Mattinata, Margherita Di Savoia, Trani, Bisceglie, Mola Di Bari, Bari, Monopoli, Ostuni-Costa Merlata, Fasano, Castellaneta Marina, Gallipoli, Salve, Ugento (Apulia region)* 5 *Metaponto Lido (Basilicata region)* 6 *Capopiccolo-Isola Capo Rizzuto, Isola Capo Rizzuto, Le Castella Isola Capo Rizzuto, Bagnara Calabra (Calabria region)* 7 *Pozzallo (Sicily region)* 8 *Scoglio Lungo, Fiume Santo, Lido San Giovanni, Le Bombarde (Sardinia region)* 9 *Battipaglia, Eboli, Capaccio (Campania region)* 10 *Follonica, Cecina, Pietrasanta (Tuscany region)* 11 *Lavagna (Liguria region)*
Data accessibility	*With this article*
Related research article	*Rodella I., Madau, F., Mazzanti, M., Corbau, C., Carboni, D., Utizi, K., Simeoni, U., 2019. Willingness to pay for management and preservation of natural, semi-urban and urban beaches in Italy. Ocean Coast Manag 172,93:104.*https://doi.org/10.1016/j.ocecoaman.2019.01.022[1]

**Table:** 

**Value of the data** • WTP literature review may be useful as reference data for future studies on economic value of beaches; • These data could be useful in comparing beach classifications of Mediterranean beaches; • Data were collected in such a way as to obtain beach users' willingness to pay (WTP). These data may thus be useful to researchers comparing users' WTP in different beach typologies.

## 1 Data

This dataset presents information on: WTP studies in coastal management, Italian beach characteristics and classifications, WTP and some demographic beach users’ statistics. Beach characteristics are used as variables in a multivariate model of WTP [1].

Supplementary material 4 presents a literature review about WTP applications in coastal management.

**Fig. 1:**
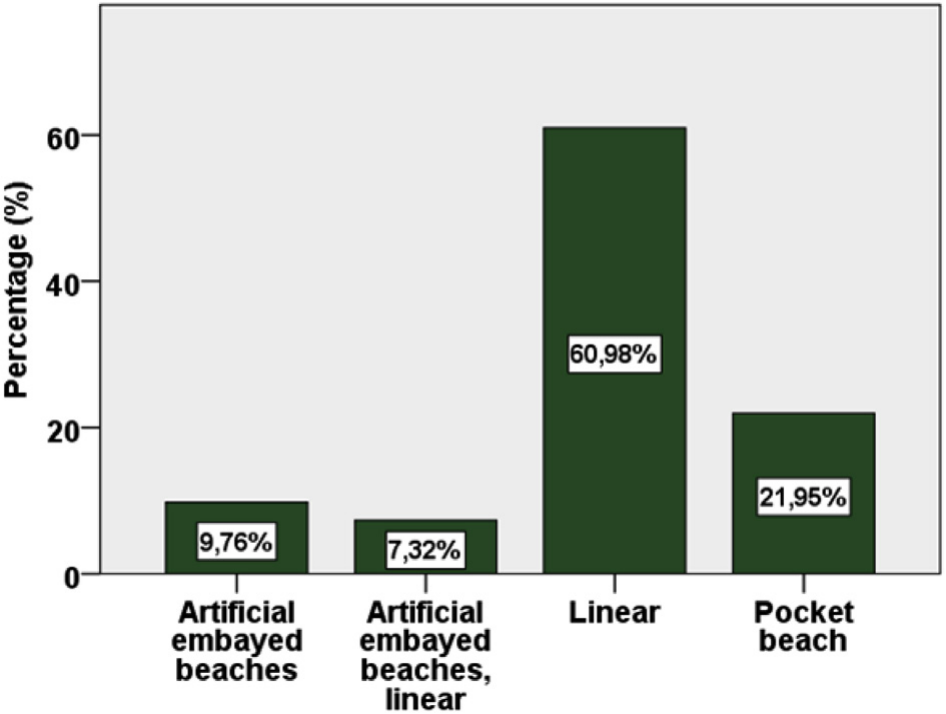
Selected beaches classified by shape.

**Fig. 2:**
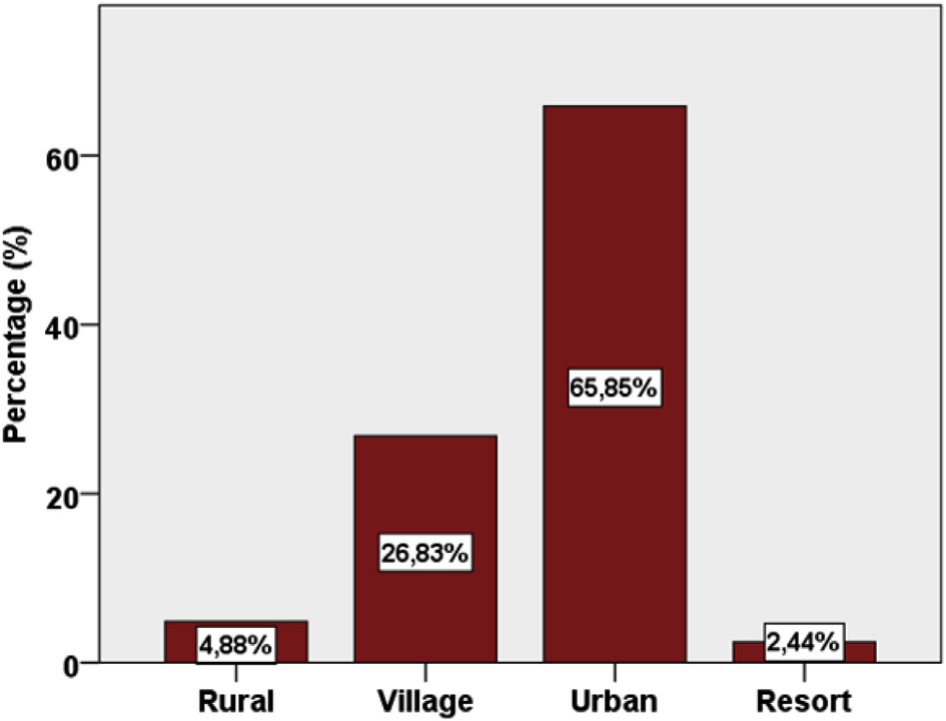
Selected beaches classified by anthropogenic classification [2].

**Fig. 3:**
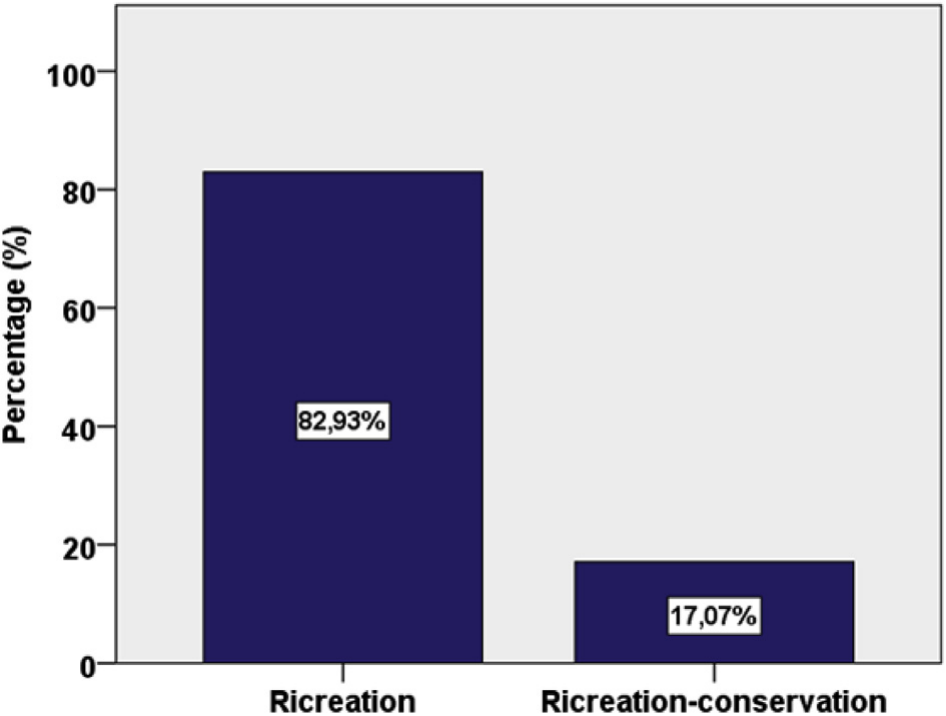
Selected beaches classified by activity classification [2].

**Fig. 4:**
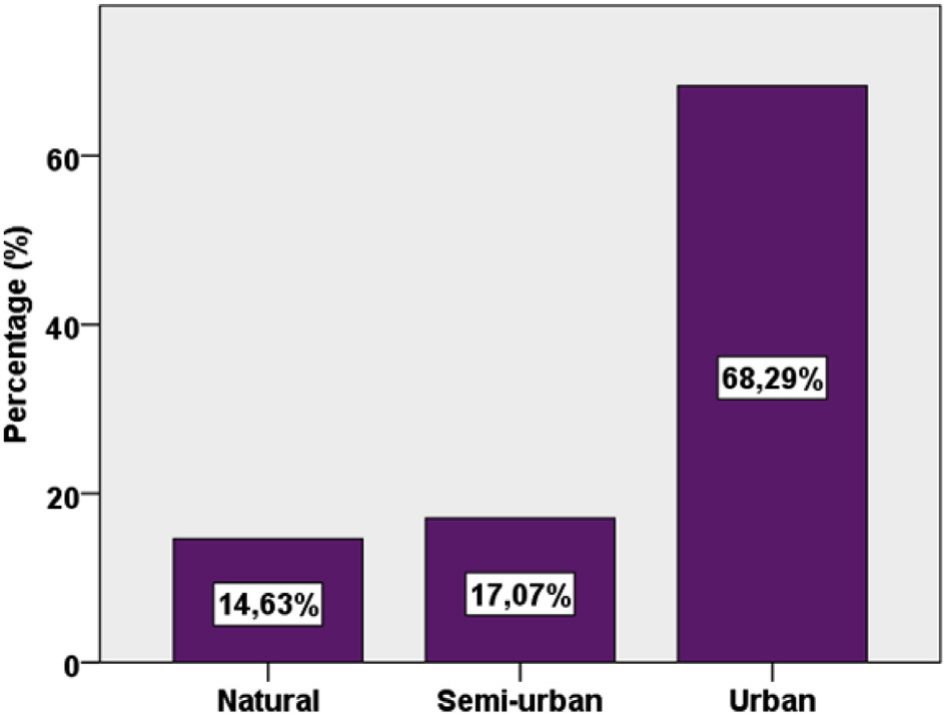
Selected beaches classified by Ref. [1].

**Fig. 5:**
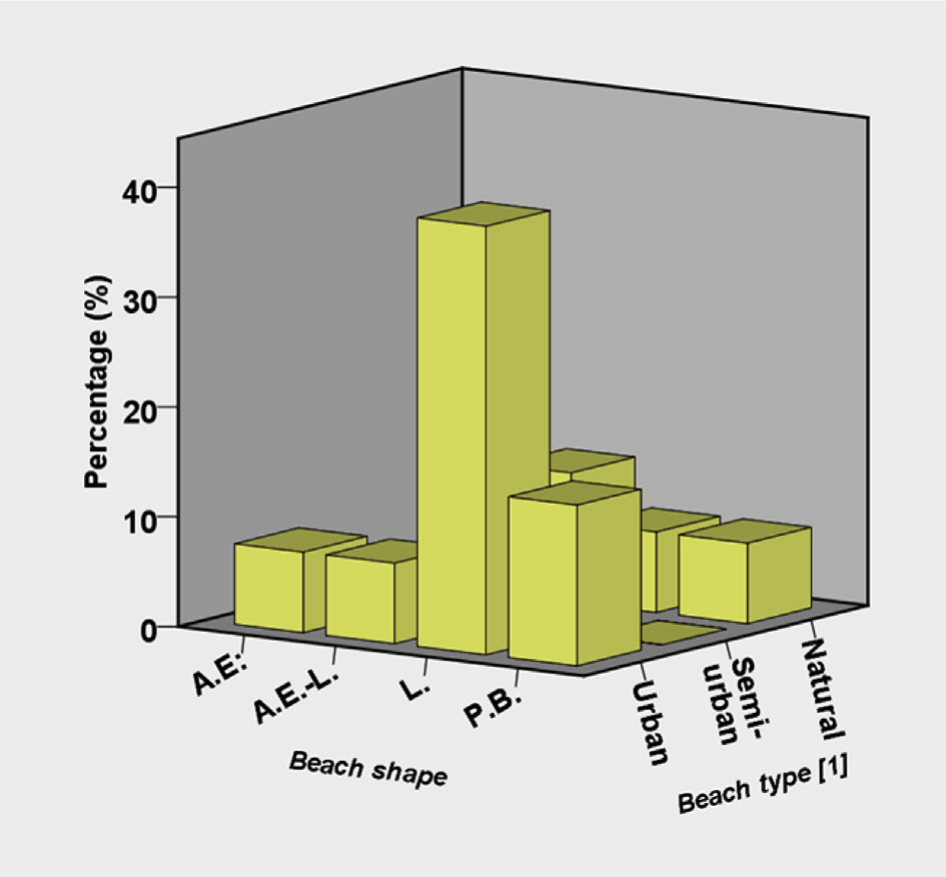
Correspondence between beach shape and beach use characteristics (A.E.: artificial embayed beach; A.E.-L.: artificial embayed beach-linear; L.: linear beach; P.B.: pocket beach).

**Fig. 6:**
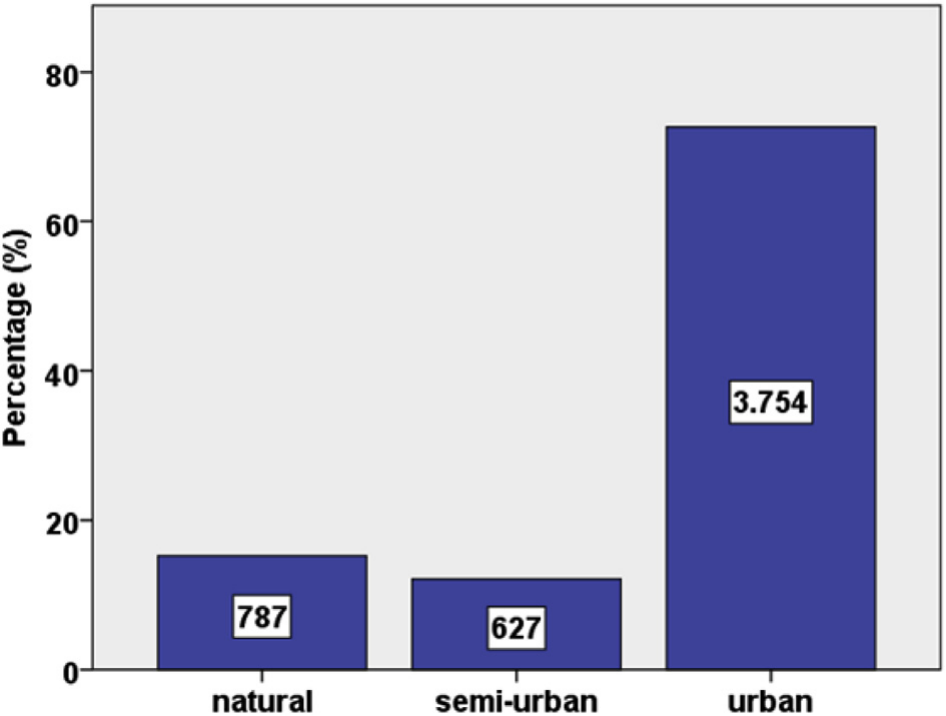
Questionnaire distribution in different beach typology defined by Ref. [1].

Supplementary material 2, from Fig. 1, Fig. 2, Fig. 3, Fig. 4, Fig. 5 graphs report data about beach characteristics and typologies of the case studies. Fig. 6 shows the questionnaires distribution for each beach type. The results from this dataset are presented in Ref. [1].

**Fig. 7:**
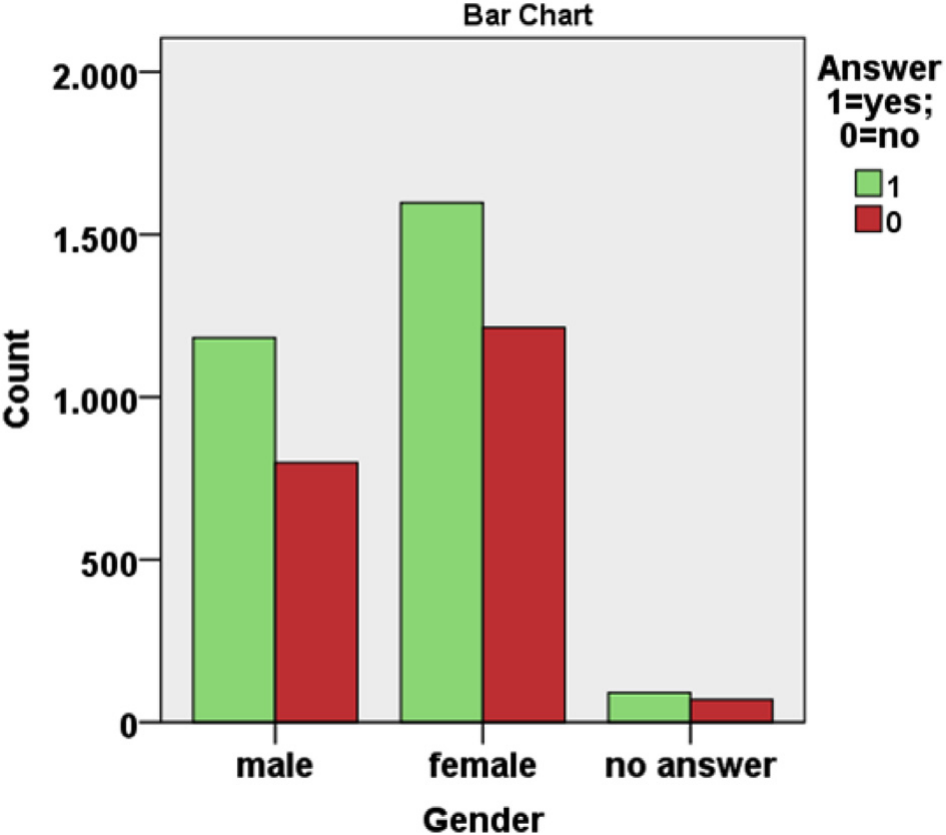
Answer to initial BID 0 related to gender.

**Fig. 8:**
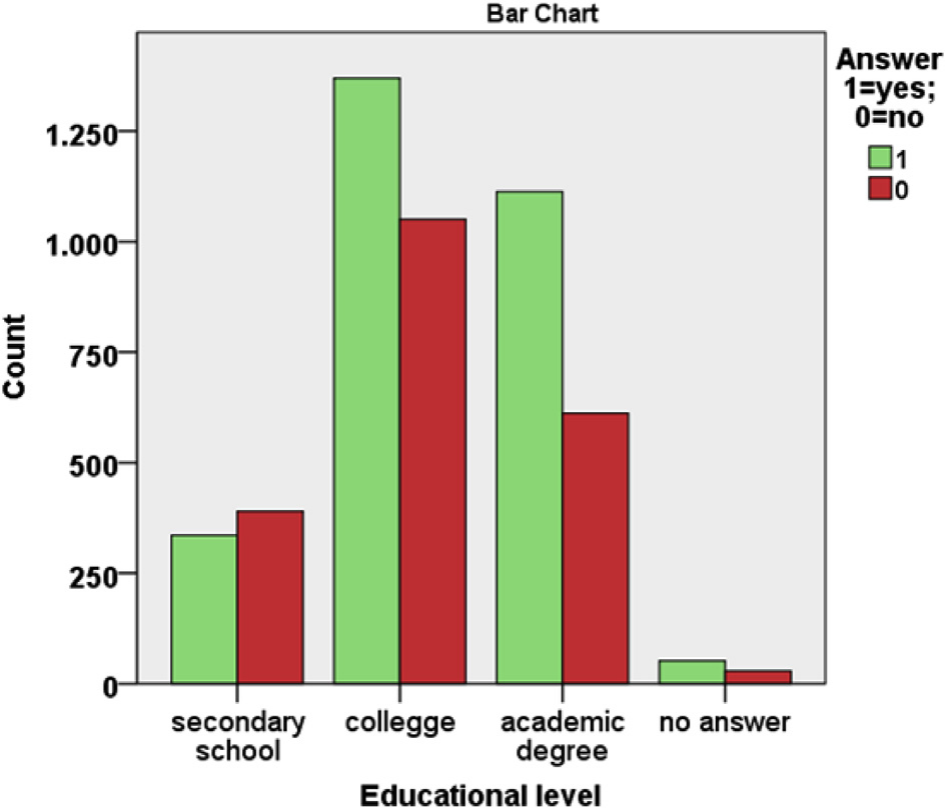
Answer to initial BID 0 related to educational level.

**Fig. 9:**
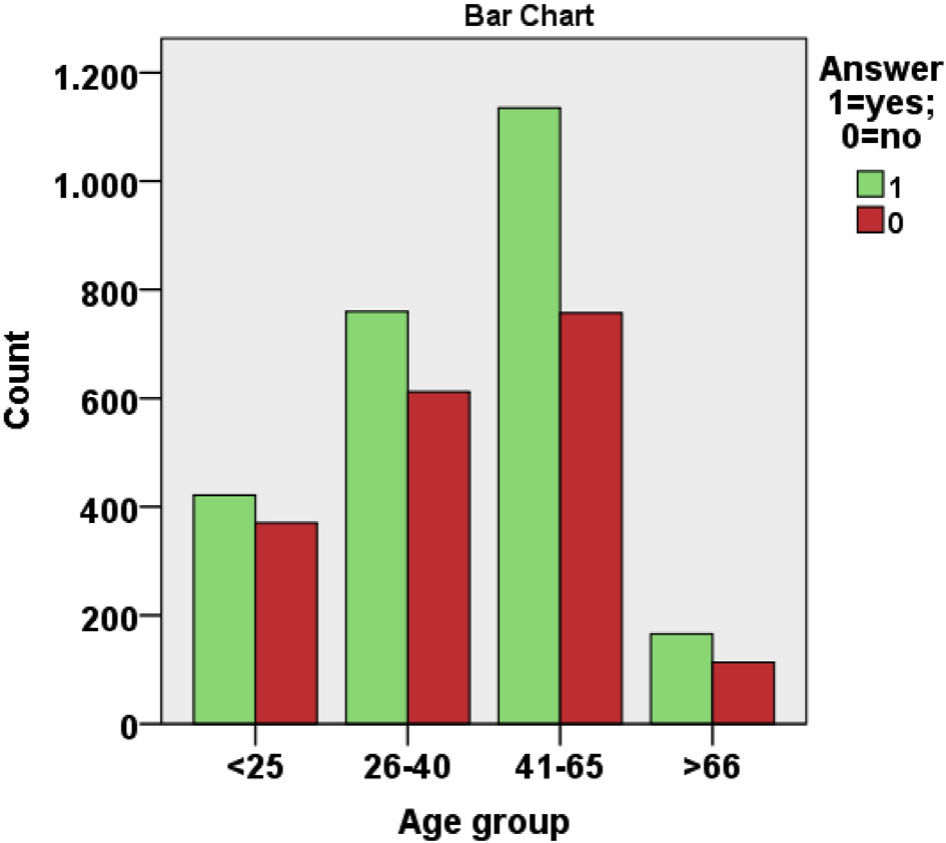
The percentage of answer to initial BID 0 related to beach users age.

**Fig. 10:**
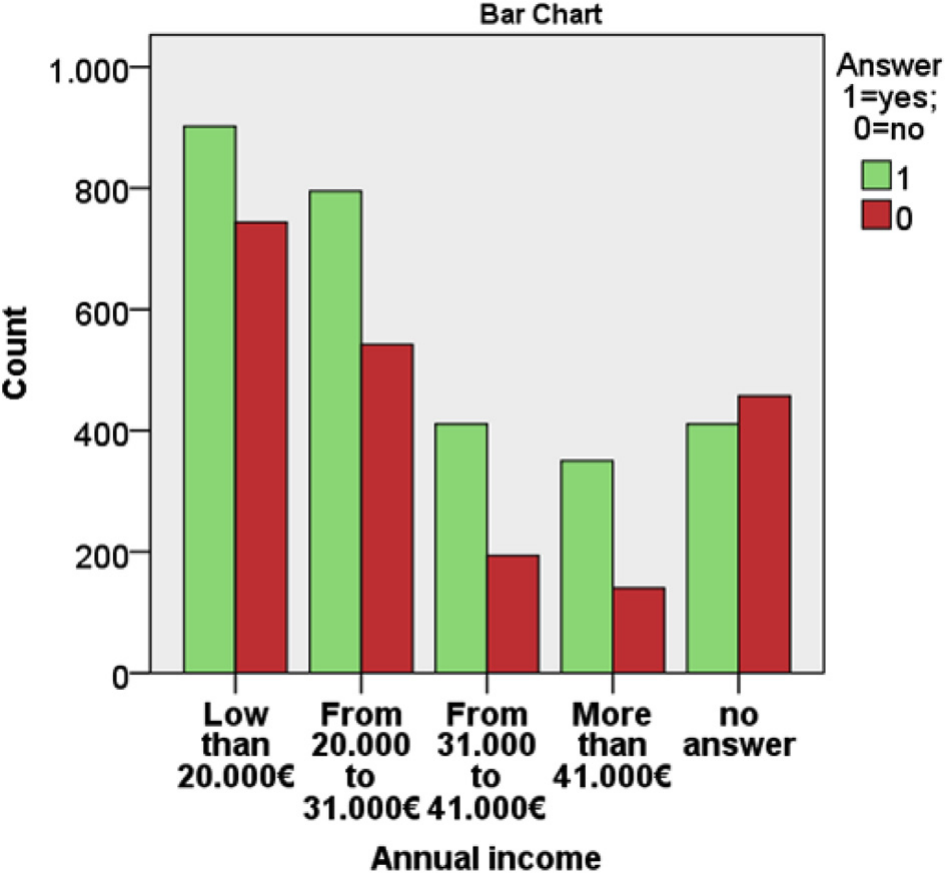
The percentage of answer to initial BID 0 related to annual income of beach users.

**Fig. 11:**
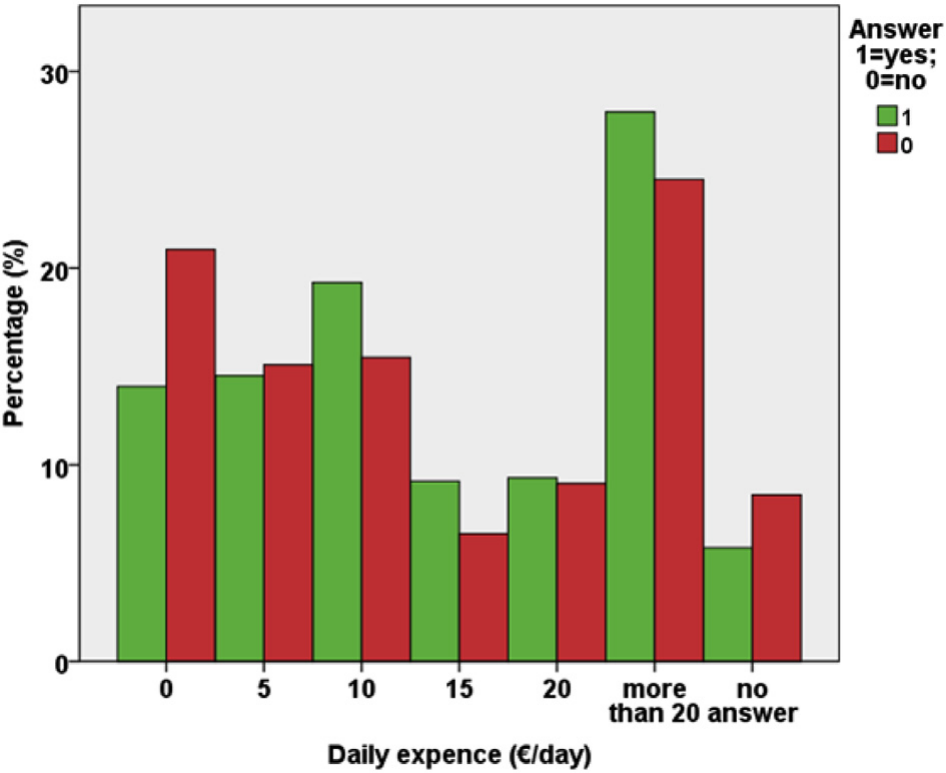
Answer to initial BID 0 related to daily expense of beach users.

From Table 1, Table 2, Table 3 and from Fig. 7, Fig. 8, Fig. 9, Fig. 10, Fig. 11 data present correlations between WTP and some demographic characteristics of beach users.

**Table 1:** Distribution of WTP response in the DB CVM:BID 0 (Pearson chi-square value = 176,857; degree of freedom = 4; p-value = 0.000).

BID 0 (€)	Yes (%)	No (%)	Total (%)
0	0.3	0.2	0.5
2	17.5	8.1	25.5
5	15.8	8.7	24.4
10	13.0	10.7	23.8
20	11.4	14.3	25.8
Total	58.0	42.0	100

**Table 2:** Distribution of WTP response in the DB CVM: BID 1 (Pearson chi-square value = 682,754; degree of freedom = 28; p-value = 0.000).

BID 1 (€)	Yes (%)	No (%)	Total (%)
1	1.5	6.6	8.1
2.5	1.9	6.7	8.6
4	12.7	5.2	17.9
5	3.2	7.7	10.9
10	13	16.8	29.8
20	5.2	7.6	12.7
40	3.3	8.2	11.5
Total	40.8	58.7	99.5

**Table 3:** Results of resident and tourist WTP (Pearson chi-square value = 6.377; degree of freedom = 2; p-value = 0.041).

Answer	% Per category	Residents WTP (%)				Tourists WTP (%)			
		Natural	Semi-urban	Urban	Total	Natural	Semi-urban	Urban	Total
Yes	% in Beach use classification	18.0	13.4	18.2	17.5	45.7	40.0	36.5	38.1
	% of the total answer	2.1	1.9	13.5	17.5	5.3	5.6	27.1	38.1
No	% in Beach use classification	8.9	7.4	13.7	12.3	25.0	35.4	27.1	28.0
	% of the total answer	1.0	1.0	10.2	12.3	2.9	5.0	20.1	28.0
No answer	% in Beach use classification	73.0	79.2	67.0	69.4	29.3	24.6	36.1	33.7
	% of the total answer	8.5	11.1	49.8	69.4	3.4	3.4	26.8	33.7

## 2 Experimental design, materials and methods

Researchers collected these data by in-person collection of questionnaires from visitors and residents to Italian beaches (Interactive map data). A questionnaire was used to elicit visitor preferences and willingness to pay for coastal preservation. The questionnaire was based on those used by Refs. [3], [4] and was structured in sections (Supplementary material 3). Data were collected from June to September 2015. Only people over 16 years old were randomly selected and interviewed. In the case of a group visit, one person was interviewed in order to avoid the risk of doubling answers. They were also informed that there was no right or wrong answer and their sincere responses would be appreciated [1], [5]. Presents single case studies of Italian beaches, while in this manuscript the overall national point of view is elaborated.

Statistical and descriptive analyses of WTP surveys were performed using the Statistical Package for Social Sciences (SPSS) version 20 (Statistics Solutions) and Microsoft Excel version 2017 (Microsoft Office, Redmond, Washington, USA).

## Appendix ASupplementary data

Supplementary material 2 Main beach characteristics and typologies. Supplementary material 2

Supplementary material 3 The questionnaire. Supplementary material 3

Supplementary material 4 Literature review about WTP in coastal management. Supplementary material 4

Supplementary material 5 Interactive map data. The selected beaches (kmz file). Supplementary material 5
