# Supplementary material for: Data for the analysis of willingness to pay for Italian beaches
Source: Data Brief. 2019 Mar 7;23:103815. doi: 10.1016/j.dib.2019.103815 (PMC6661251; doi:10.1016/j.dib.2019.103815)
Supplement: Supplementary material 3 — The questionnaire. [file mmc3.doc]

| **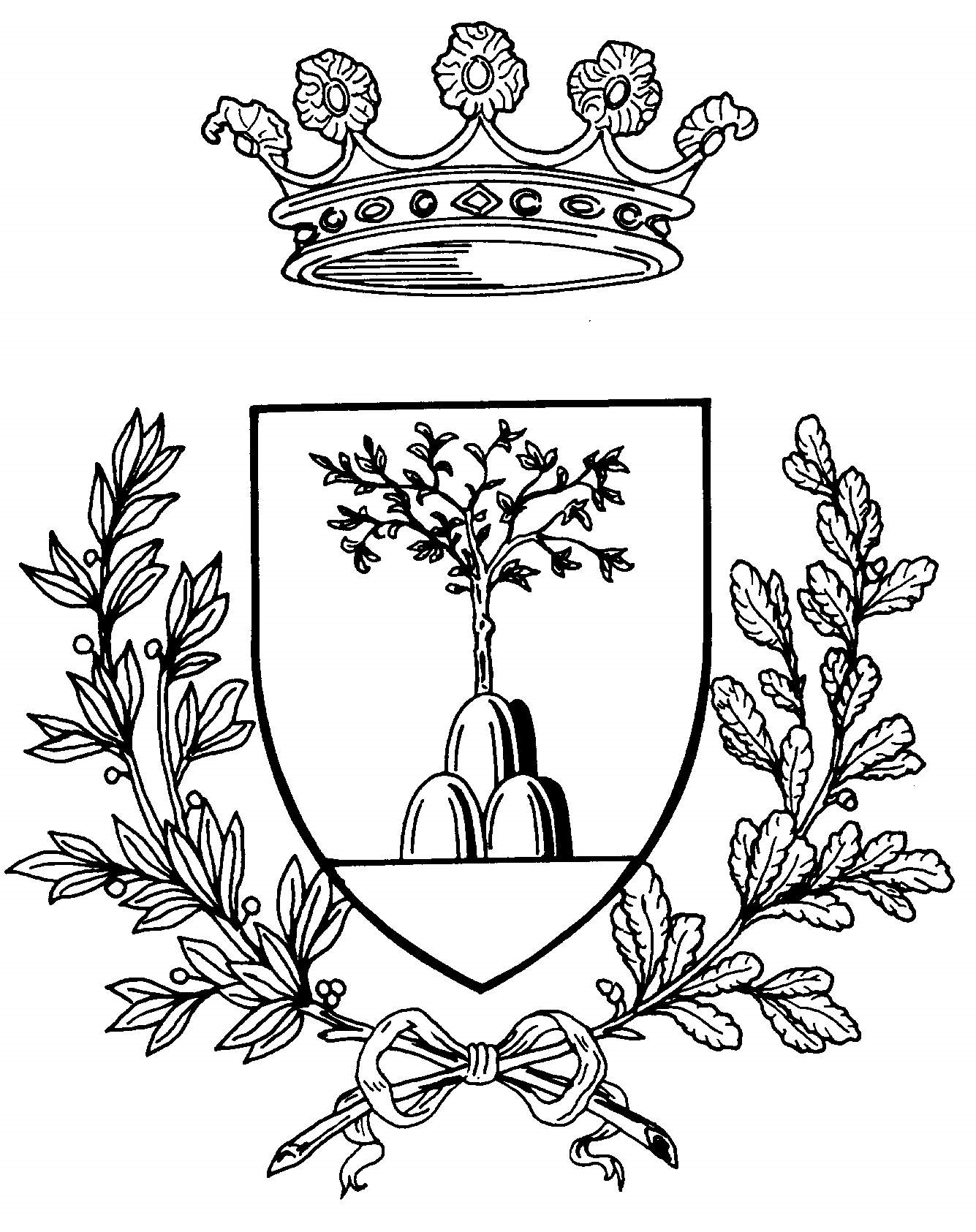** | **DIPARTIMENTO DI FISICA E SCIENZE DELLA TERRA - UNIVERSITA’ DI FERRARA**  Via G. Saragat, 1 Blocco B – 44100 FERRARA (ITALY)  TEL. 39-0532-974694 - FAX. 39-0532974647 |
| --- | --- |

# BEACH TOURIST’S PERCEPTION ANALYSIS

The survey aims to investigate the beach users’ preferences: expectations, the beach value and characteristics, knowledge of environmental issues and criticities.

The questionnaire is anonymous, voluntary, confidential and will be used exclusively for research purposes.

**QUESTIONNAIRE TO TOURISTS AND RESIDENTS**

**GENERAL INFORMATIONS:**

**Date**………………………………………..**Municipality of**…………………………………………..

**Beach/resort infrastructure** (indicate if you’re in a free beach or resort infrastructure) ……..……………………………………………………………………………………

**Gender:**  M  F **Age:**……………

**Qualification**:

 secondary school

 college

 academic degree

**Provenience:**

 resident

 not resident (indicate your origin):

city…………….………………………nationality………………………………….

1. **Is it the first time you come to this resort?**

 yes

 no, I come habitually

 no, I’ve already been here sometimes

1. **Are you satisfied of your holiday?**  yes  no
2. Who are you here with**?**

 alone  partner  family (with sons)  friends  someone else

1. **What is the main reason why you have chosen this resort?** (only one box)

|  sea/beach |  play sport/amusement |
| --- | --- |
|  nature and landscape |  relax/quiet |
|  cultural heritage (handicraft/folklore/cooking) |  have a holiday home |
|  economic reasons | other (specify)………………………………….. |

1. **How many days will you remain in this resort?**

 only today  from 2 to 7  from 8 to 15  more than 15

1. **How many hours on the average do you spend daily at the beach?**

 less than 2  from 2 to 6  from 6 to 10

1. **Which features do you prefer better at a beach?** (choose not more than three box )

|  clean sea |  services/facilities (bar, showers, beach huts, etc.) |
| --- | --- |
|  clean beach |  swimming pool |
|  safety |  sport/recreational activities |
|  landscape |  other |

1. **Which assessment do you give to the sea and the beach where you are at this moment?**

| • beach quality and cleanness |  good  sufficient  low |
| --- | --- |
| • water quality |  good  sufficient  low |
| • beach width |  excessive  adequate  insufficient |
| •crowding |  high  high only on the week-end  medium  low |
| • safety |  good  sufficient  low |
| • recreational activities |  good  sufficient  low |
| • services |  good  sufficient  low |
| • landscape | beautiful  indifferent  bad |

1. **Which of the following scenarios would you prefer? (choose 1 box)**

| 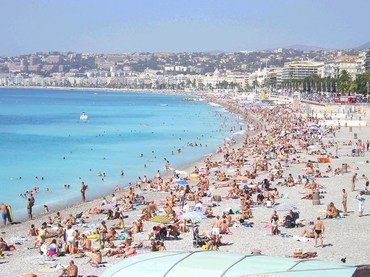 | 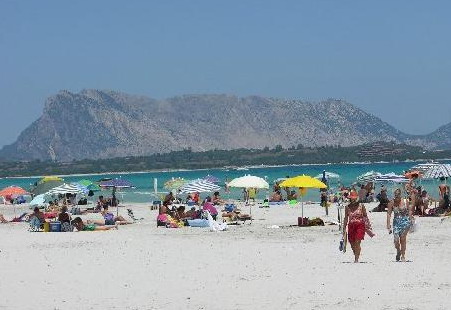 |
| --- | --- |
| 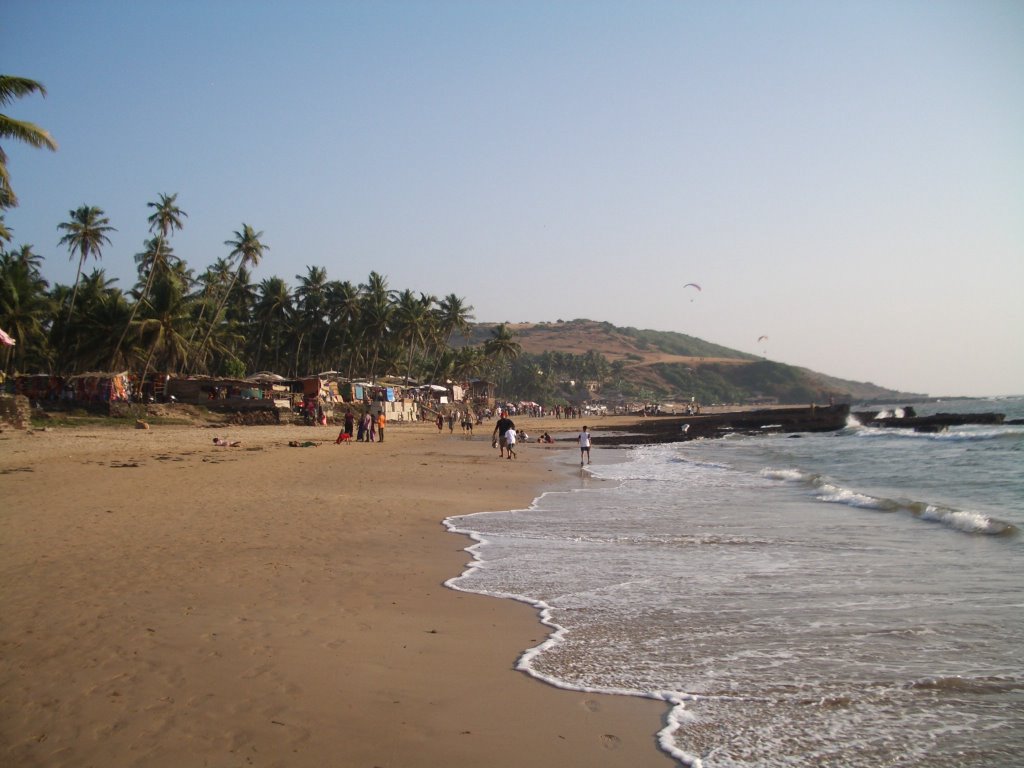 | *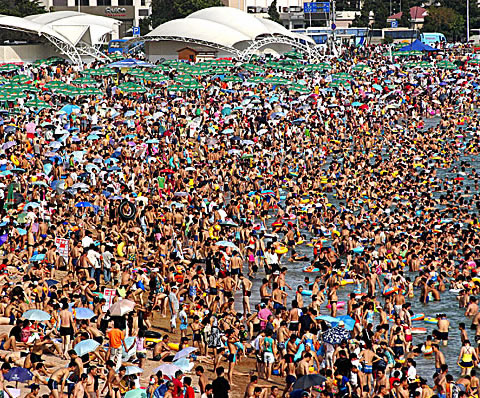* |

1. **How do you evaluate the available beach space for yourself?**

 *adequate,*  *sufficient,*   *insufficient*

**In your opinion, what space per person is adequate?**

 *4 m^2^,*  *8 m^2^,*  *10 m^2^, other___________*

1. **What value do you attribute to landscape?**  high  medium  low
2. **How do you prefer the free beach?**

 not equipped and cost-free  equipped with a small expense for the services

1. **In your opinion, the beaches are:**  a public good  a private good
2. **Do you know what is “the beach erosion”?**  yes  no
3. **In your opinion, is the beach erosion an important problem?** yes  no
4. **What is your tax bracket?**

 low than 20.000 €  from 20.000 to 31.000 €  from 31.000 to 41.000 €  more than 41.000 €

1. ***In case that a financial fund is constituted in order to ensure the appropriate management of beach:***

- **FOR THE TOURISTS: Would you pay 2 € (for person) each season in this territory?**

 yes  no

- **FOR THE RESIDENTS: Would you pay 2 €/year (for person)?**

 yes  no

- *If YES, would you also pay 4 €?*   yes  no
- *if NO, would you pay instead 1 €?*   yes  no

1. **How much do you normally spend every day at the beach?** *(€/person)________________*

*(considering umbrella, facilities, restaurant, coffee bar etc..)*

1. **Do you know what is the “Integrate Coastal Zone Management” (ICZM)?**  yes  no
2. **Do you know any of the following coastal protection techniques**?

| **1 -** Groynes  yes  no | **2 -**  Breakwaters  yes  no |
| --- | --- |
| **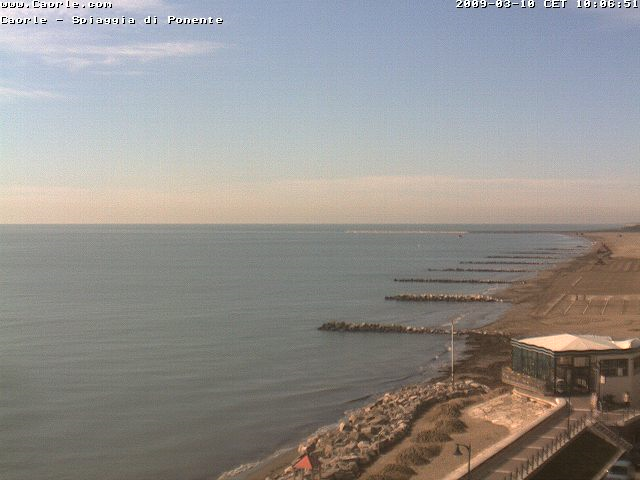** | **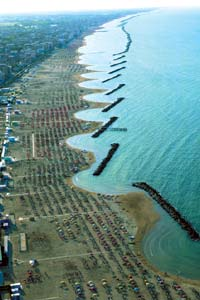** |
| **3 -** Mixed interventions  yes  no | **4** **-** Submerged barriers  yes  no |
| **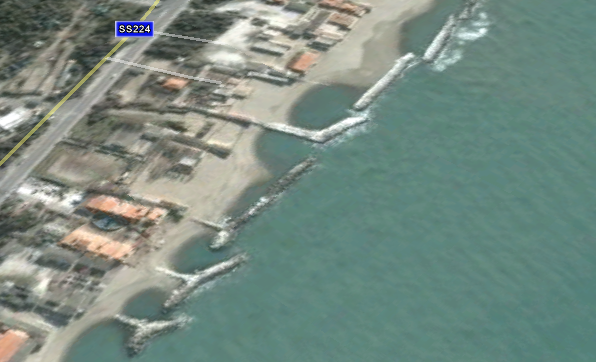** | **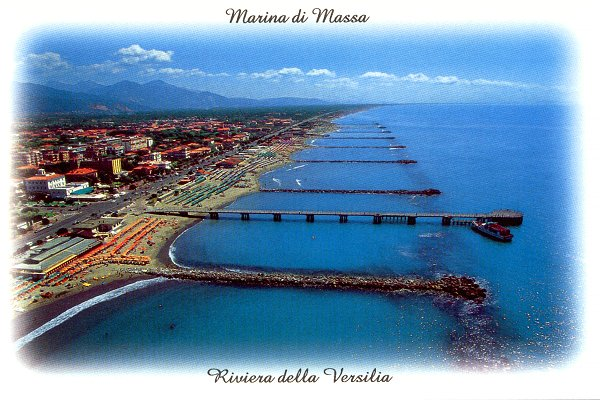** |

*In your opinion, what is the most effective protection system?*  **1**  **2**  **3**  **4**

*In your opinion, what is the protection system that has the minor impact on the environment?*  **1**  **2**  **3**  **4**

1. **. Do you know what is a “beach nourishment” ?**  yes  no

*If yes, in your opinion, what is it?*

*………………………………………………………………………………………*

1. **Do you think that beach nourishment is necessary for the beach?**  yes  no

**OBSERVATIONS**

………………………………………………..…..…………………………………………………………………………….………………………………………………………………………………………………………………………………….……………………………………………………………………………………………………………………………….
